# Supplementary figures and images for: Cuproptosis-associated ncRNAs predict breast cancer subtypes
Source: PLoS One. 2024 Feb 26;19(2):e0299138. doi: 10.1371/journal.pone.0299138 (PMC10896520; doi:10.1371/journal.pone.0299138)

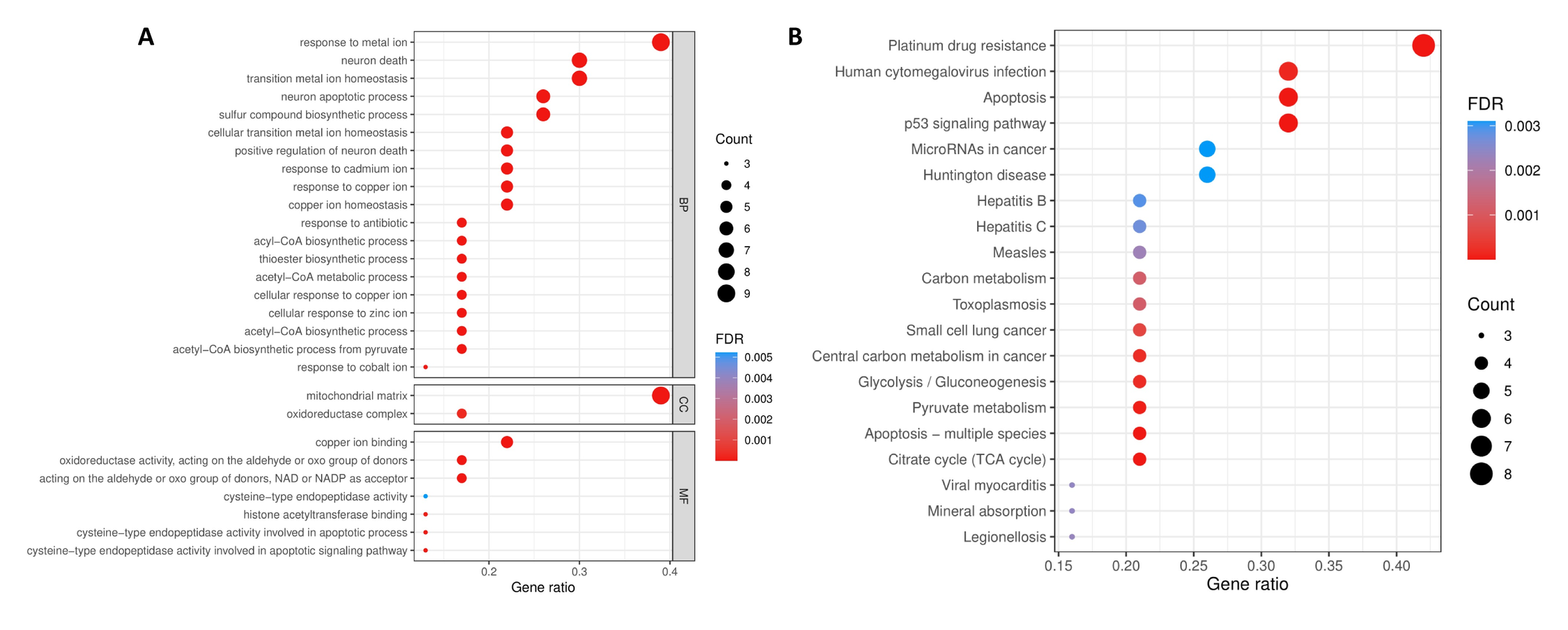

Supplement: S1 Fig — (TIF) [file pone.0299138.s001.tif]

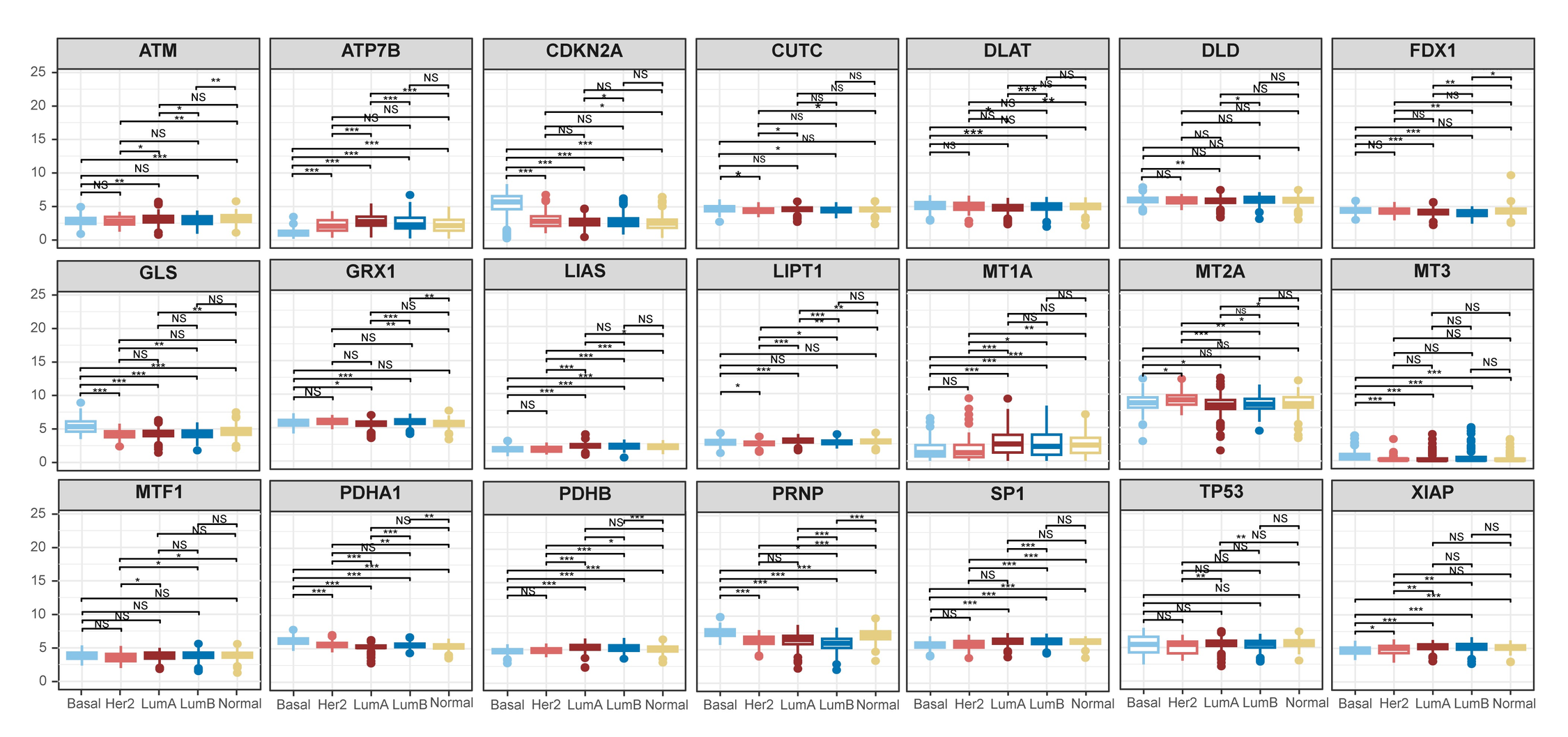

Supplement: S2 Fig — (TIF) [file pone.0299138.s002.tif]
