# Supplementary material for: Cuproptosis-associated ncRNAs predict breast cancer subtypes
Source: PLoS One. 2024 Feb 26;19(2):e0299138. doi: 10.1371/journal.pone.0299138 (PMC10896520; doi:10.1371/journal.pone.0299138)
Supplement: Attachment — Submitted filename: Response to Reviewers.docx [file pone.0299138.s011.docx]

**Response to review for PONE-D-23-17810:** ***Cuproptosis-associated ncRNAs predict breast cancer subtypes***

Dear Editor and Reviewers:

Thank you for your thoughtful review of our manuscript and the opportunity to further improve our work by revision. We have made changes according to the suggestions by the editors and reviewers, and trace marked the revised part in our manuscript. Please find our point-by-point response to all the inquiries and updated tables or figures below.

**Reviewer #1**

**Comment 1: Breast cancer epidemiology needs to be updated.**

**Response**: Thank you for your suggestions. Published by the World Health Organization in 2020, "Global Cancer Statistics 2020: GLOBOCAN Estimates of Incidence and Mortality Worldwide for 36 Cancers in 185 Countries" represents the most up-to-date global cancer epidemiology data. Furthermore, we have included the most recent incidence figures for breast cancer in the United States as of 2023. Please check the tracked changes for our revisions.

The specific modifications are as follows:

- On Page 4, Line 50:

Based on projections by the American Cancer Society, it is anticipated that there will be approximately 288,000 new cases of breast cancer diagnosed in the United States in 2023, with an estimated 43,000 individuals succumbing to the disease (PMID: 36633525).

**Comment 2: ncRNAs have a new classification criterea. Update**

**Response:** Thank you for your suggestions.

We have revised the classification of ncRNAs and incorporated the tracked changes for your reference.

The specific modifications are as follows:

- On Page 4, Line 72:

The classification of ncRNAs based on function and nucleotide length (nt) has been refined as follows: ncRNAs with a length less than 200 nt, such as microRNA (miRNA), transfer RNA (tRNA), and small interfering RNA (siRNA). ncRNAs with a length exceeding 200 nt, encompassing Ribosomal RNA (rRNA), long non-coding RNA (lncRNA), and circular RNA (circRNA) (PMID: 33860799).

**Comment 3: It's not clear why in the objective they focus on ncRNA and in the title in lncRNAs**

**Response:** Thank you for your suggestions.

In light of the discovery that the crucial genes we have identified primarily fall under the category of lncRNA, we have made a swift adjustment to our article title. The title has been modified to "Cuproptosis-associated ncRNAs predict breast cancer subtypes." We acknowledge that our initial choice of title, "Cuproptosis-associated lncRNAs predict breast cancer subtypes," was an oversight.

**Comment 4: Maybe logFC value >|1| is too high for a molecule with several regulatory roles.**

**Response**: Thank you for your suggestions. We have incorporated the tracked changes for your revisions, as specified below:

- On Page 7, Line 116：

Significant differential expression was defined as a false discovery rate (FDR) < 0.05 and an absolute value of log fold-change (|logFC|) ≥ 2.

- On Page 12, Line 215:

A total of 194 differentially expressed ncRNAs were identified in breast cancer, comprising 52 up-regulated and 142 down-regulated ncRNAs. Subsequently, Pearson correlation analysis was employed to identify 94 ncRNAs significantly correlated with Cuproptosis.

We utilized the random forest algorithm to assess the importance of the 94 ncRNAs associated with Cuproptosis. The top five genes, with the highest relative importance values, were selected as key genes and integrated into the final model.

**Comment 5: 5. Is not clear why immune profile was evaluated.**

**Response**。Please check the tracked changes for our revisions. We have included a rationale for evaluating immune profiles due to their critical importance in tumor immunotherapy. Here are the specific modifications:

- On Page 8, Line 136:

The tumor immune microenvironment consists primarily of tumor cells, fibroblasts, immune cells, and an array of signaling molecules. It is well-established that the tumor microenvironment plays a pivotal role in influencing tumor diagnosis, survival outcomes, and clinical treatment responsiveness (PMID: 36341428). Thus, gaining insights into the composition of immune cells within tumors is indispensable for advancing cancer treatment strategies.

**Comment 6: Results discussion needs to be improved.**

**Response**: Thank you for your suggestions. We have reviewed the tracked changes for your revisions. Here are the specific modifications:

- On Page 16, Line 324:

m1A modification encompasses a range of critical effects on RNA, influencing processes such as RNA processing, secondary and tertiary structure, stability, translation efficiency, and biological function (PMID: 36467585). A pivotal contributor to m1A modification is TRMT61B, a mitochondria-specific tRNA methyltransferase primarily responsible for installing m1A (PMID: 23097428) at position 58 (m1A58) of tRNA. Additionally, TRMT6 and TRMT61A have been implicated in glioma, gastrointestinal cancer, and hepatocellular carcinoma (HCC) development (PMID: 35444240, 35444240, 35444240). Notably, ALKBH1, exhibiting demethylase activity, catalyzes the demethylation of m1A modification (PMID: 37531210).

The m5C modification, on the other hand, is intimately involved in the progression of various tumors. NOP2/Sun domain family members 1-7 (NSUM 1-7) and the DNMT homolog DNMT2 serve as m5C writers in mammals, orchestrating the methylation of the RNA C5 position (PMID: 16424344). Conversely, TET2 acts on m5C, converting it into 5-hmC and eventually removing the methyl group (PMID: 29364877). Furthermore, ALYREF functions as a reader, specifically recognizing and binding to the m5C motif, thereby exerting additional biological functions (PMID: 28418038).

Finally, m6A modification plays a vital role in governing mRNA stability, splicing, degradation, and translation efficiency (PMID: 32398132). The generation of m6A modification hinges on the methyltransferase complex, consisting of METTL3/METTL14/WTAP proteins. Concurrently, YTHDC1/2, YTHDF1/2/3, IGF2BP1/2/3, and HNRNP act as readers, proficient in recognizing m6A residues (PMID: 27558897).

- On Page 18, Line 371:

In this study, we employed a random forest model to discern five breast cancer subtype biomarkers linked to cuproptosis. Among these, four lncRNAs (CASC8, GRIK1-AS1, LINC02188, and LINC02095) exhibit diminished expression levels in breast cancer, while one lncRNA (LINC00511) displays elevated expression in breast cancer. Notably, CASC8, LINC02095, LINC02188, and LINC00511 exhibit increased expression within the Basal and Normal subtypes. Conversely, GRIK1-AS1 shows elevated expression in the Luminal A (Lum A) subtype.

Previous studies have underscored the significance of these lncRNAs in breast cancer biology. LINC02095 has been implicated in promoting breast cancer proliferation by facilitating the expression of the oncogenic transcription factor SOX9 (PMID: 31690584). CASC8 has demonstrated associations with the risk of several cancers, including non-small cell lung cancer, retinoblastoma, esophageal squamous cell carcinoma, and colorectal cancer, and has been found to be highly expressed in hepatoma cells like MIA PaCa-2. LINC00511 may upregulate the expression of MMP13 by acting as a miR-150 sponge, thereby promoting breast cancer cell proliferation, migration, and invasion (PMID: 33031905). GRIK1-AS1 serves as a sponge for miR-375, leading to increased IFIT2 protein levels and the promotion of gastric cancer progression (PMID: 34660323). Additionally, GRIK1-AS1 can impede DNMT1 from binding to the SRFP1 promoter, resulting in SFRP1 hypomethylation and subsequent upregulation, thereby accelerating the progression of endometriosis (PMID: 37392032). Presently, limited studies have explored the functions of LINC02188. These findings underscore the close associations between most of the key genes and the onset and progression of cancer. They assume distinct roles in the development and advancement of tumors within different subtypes. As such, they hold the potential for use as subtype identification markers and are prospective targets for future breast cancer treatments.

- On Page 20, Line 416:

Previous research has delved into cell death patterns concerning breast cancer, with a particular focus on copper death. For instance, a study demonstrated that regulators associated with copper-induced cell death can serve as prognostic indicators, predict chemotherapy response, and anticipate immunotherapy outcomes in breast cancer patients (PMID: 36226193). Deng et al. harnessed machine learning techniques to identify copper death-related genes, ultimately constructing a novel ceRNA network and risk model tailored to breast cancer (PMID: 36980514). Li et al. devised a copper death scoring system aimed at forecasting tumor microenvironment infiltration characteristics and gauging the efficacy of immunotherapy (PMID: 36212436). These investigations have primarily concentrated on leveraging copper death mechanisms to forecast the prognosis of breast cancer patients.

In contrast, our study pioneers a novel approach by formulating a breast cancer subtype prediction model grounded in copper death-related ncRNAs. The five key genes utilized in constructing this model exhibited distinct expression patterns across various breast cancer subtypes. This innovative approach offers fresh perspectives for tailoring personalized treatments for breast cancer patients.

Finally, we appreciate for Editors/Reviewers’ warm work earnestly, and hope that the correction will meet with approval. Once again, thank you very much for your comments and suggestions.

Best wishes,

Yours sincerely,

Shiwei Duan, Ph. D, Professor

School of Medicine,

Hangzhou City University,

Zhejiang, China

Email: duansw@hzcu.edu.cn
