# Supplementary material for: Cuproptosis-associated ncRNAs predict breast cancer subtypes
Source: PLoS One. 2024 Feb 26;19(2):e0299138. doi: 10.1371/journal.pone.0299138 (PMC10896520; doi:10.1371/journal.pone.0299138)
Supplement: Attachment — Submitted filename: Response to Reviewers_cuproptosis.docx [file pone.0299138.s012.docx]

**Response to review for PONE-D-23-17810:** ***Cuproptosis-associated ncRNAs predict breast cancer subtypes***

**Dear Editor and Reviewers:**

Thank you for your thoughtful review of our manuscript and the opportunity to further improve our work by revision. We have made changes according to the suggestions by the editors and reviewers, and trace marked the revised part in our manuscript. Please find our point-by-point response to all the inquiries and updated tables or figures below.

**Reviewer #1**

**Comment 1: does SOX9-AS1 increase or decrease the prediction accuracy in Lum B subtype.**

**Response**: In response to the feedback from reviewer 1, who noted that the screening conditions were overly broad in the initial revision, we have made significant adjustments to the study. Specifically, we refined the screening conditions to focus on genes with a |logFC| value greater than 2 and subsequently conducted a re-screening of key genes. Notably, our reevaluation has led to the exclusion of SOX9-AS1 from the model establishment as a key gene, a departure from its prior inclusion with a contribution of 0 to predicting the Lum B subtype in previous studies. The updated findings are elucidated in the following Fig. 4D, where genes that do not manifest are assigned a zero contribution. Importantly, such genes neither augment nor diminish the accuracy of our predictive model, as visually represented in the figure. This adjustment reflects our commitment to refining the precision and relevance of our study, addressing concerns raised by the reviewers, and ensuring the robustness of our predictive model.

**Fig. 4. Validation of the cuproptosis-related model and the role of key ncRNAs in the model**

**Comment 2: Do the authors check the correlation between ncRNAs and checkpoint molecules? This will be good information.**

**Response:** Thank you for your suggestions. We have conducted correlation studies investigating the relationship between ncRNAs and checkpoint molecules. Our findings reveal a positive correlation between three pivotal genes and eight immune checkpoint genes, namely CD96, CTLA4, LAG3, PD-1, PD-L2, PD-L1, TIGIT, and VTCN1. The specific modifications are as follows:

- On Page 9, Line 158:

Immune checkpoints play a pivotal role as crucial molecules employed by the immune system to finely tune the expression of its own proteins. Tumor cells, adept at evading immune responses, often achieve this evasion through the intricate regulation of immune checkpoints (PMID:28782469). To delve into the association between cuproptosis-related ncRNAs and immune checkpoints, we meticulously gathered data on 20 immune checkpoints from the TISIB website. Employing Pearson correlation analysis, we sought to unravel the nuanced relationship between these ncRNAs and the intricate network of immune checkpoints.

- On Page 17, Line 337:

To investigate the intricate connection between cuproptosis-associated ncRNAs and the phenomenon of immune escape, we conducted a comprehensive correlation analysis to assess the interrelation of key genes with immune checkpoints. The results, depicted in Fig. 4A, reveal a positive correlation between LINC0511, LINC02188, and ROCR, and eight pivotal immune checkpoint genes, namely CD96, CTLA4, LAG3, PD-1, PD-L2, PD-L1, TIGIT, and VTCN1. Furthermore, our investigation unearthed that LINC02188 exhibits the highest expression levels within the Basal subtype, implying a heightened vulnerability to immune escape among patients characterized by the Basal subtype, as substantiated by prior research findings.

**Comment 3: The quality of the figures is not good. Increase the image resolutions.**

**Response:** Thank you for your suggestions. We meticulously refined the image, incorporating the discussion outcomes into Fig. 1. Additionally, we restructured the visual arrangement of Fig. 4 for improved clarity. Notably, Fig. 5 now encompasses the results of the immune checkpoint-related analysis, while Fig. 6 has been enriched with the findings from the apoptosis pathway-related analysis. To enhance the visual fidelity, we have uploaded images with a higher resolution of 300 dpi to our submission system.

**Comment 4: The authors thoroughly check for typos.**

**Response**: Thank you for your suggestions. We meticulously scrutinized pertinent terms, including but not limited to "cuproptosis," "subtype," "RNA methylation modification," and "immune infiltration."

**Reviewer #2**

**Comment 1: What is the rate of survival and recurrence in breast cancer? Please include this information in introduction.**

**Response：**Thank you for your suggestions. Based on data from the Global Cancer Survival Trends Monitor, breast cancer exhibits a five-year survival rate ranging from 80% to 84% (PMID: 29395269). Furthermore, the recurrence rate within 10 to 32 years post the initial breast cancer diagnosis is reported at 16.6% (PMID: 34747484). Notably, our analysis also involved a comparson of the five subtypes, namely Basal, Her2, Lum A, Lum B, and Normal, revealing statistically significant differences in their survival rates (P<0.0001). The Lum A subtype emerged with the highest five-year survival rate at an impressive 89.5%, while the Normal subtype exhibited the lowest, with a mere 72%. Here are the specific modifications:

- On Page 4, Line 54:

According to the statistics provided by the Global Cancer Survival Trends Monitor, the five-year survival rate for breast cancer falls within the range of 80-84% (PMID: 29395269), with a recurrence rate of 16.6% within 10-32 years following the initial diagnosis (PMID: 34747484).

- On Page 12, Line 221:

Furthermore, our analysis, utilizing follow-up data from the TCGA database, delved into the survival rates across five distinct subtypes: Basal, Her2, Lum A, Lum B, and Normal. Significantly divergent survival rates were observed among these subtypes (P<0.0001). Notably, the Lum A subtype exhibited the highest five-year survival rate, reaching an impressive 89.5%. Conversely, the Normal subtype presented the lowest five-year survival rate, standing at only 72% (Fig. 3A).

**Fig. 3. Establishment of cuproptosis-related model and expression of key ncRNAs in breast cancer subtypes**

**Comment 2: What was the rationale for choosing cuproptosis related ncRNAs over cuprotosis related genes as a model for prediction. Please explain as this is not clear.**

**Response**: Thank you for your suggestions. We selected cuproptosis-related ncRNAs as the focus of our research for three compelling reasons. Firstly, our prior investigations revealed that only two cuproptosis-related protein-coding genes, namely PDHB and PRNP, exhibited significant expression differences among the five breast cancer subtypes (log2FC=1.5, P<0.001). Subsequently, we employed cuproptosis-related protein-coding genes to construct a breast cancer subtype prediction model. The model's performance on the validation set yielded an AUC of only 0.74, indicating its suboptimal predictive capabilities. Secondly, despite the absence of protein-coding capabilities, ncRNAs play a pivotal role in regulating protein production and cellular functions. They actively engage in various tumor biological processes by modulating the expression of multiple downstream genes, serving as crucial biomarkers for cancer diagnosis and treatment (PMID: 37735815). Thirdly, our investigation unveiled an unexplored correlation between breast cancer subtypes and cuproptosis-related ncRNAs. Consequently, we opted to construct a high-accuracy prediction model using cuproptosis-related ncRNAs. Noteworthy associations include lncRNA CASC8 with ATP7B, PDHB, and PRNP; LINC00511 with CDKN2A, PRNP, and the lncRNAs GLS, ROCR, and LINC02188 with PRNP and GLS; and lncRNA GRIKI-AS1 with LIPT1 and LIAS.

Here are the specific modifications:

- On Page 12, Line 228:

We identified the expression of cuproptosis-related mRNA across five distinct breast cancer subtypes, revealing that only two genes, PDHB and PRNP, exhibited significant differential expression within this cohort (Fig. S2). Employing cuprotosis-associated genes, we constructed a breast cancer subtype prediction model, unveiling an area under the curve (AUC) of 0.74 in the validation set. This result suggests suboptimal performance for the prediction model relying on cuprotosis-related genes. Subsequently, we endeavored to identify cuproptosis-related ncRNAs and establish a prediction model with superior performance.

**Fig. S2. Expression of cuproptosis-related genes in five breast cancer subtypes**

**Comment 3: Please check if canonical apoptosis pathway also has a positive correlation with the cuproptosis related ncRNAs.**

**Response**: Thank you for your suggestions. We have incorporated research findings that explore the correlation between apoptosis and ncRNAs. Here are the specific modifications:

- On Page 10, Line 186：

We compiled a set of 20 apoptosis-related protein-coding genes from relevant literature sources. Subsequently, we conducted a comprehensive analysis to elucidate the correlation between cuproptosis-related ncRNAs and the 20 apoptotic genes. Our investigation sought to uncover the distinctive roles played by these molecular components in various subtypes of breast cancer.

- On Page 18, Line 374:

This study delved into the intricate association between cuproptosis and apoptosis across various subtypes of breast cancer cells. The investigation centered on evaluating the correlation between key cuproptosis-related ncRNAs and apoptosis-related genes. The findings illuminated a noteworthy negative correlation between CASC8, ROCR, and LINC02188 with 10 apoptosis genes, as depicted in Fig. 6A. Particularly, CASC8 and LINC02188 exhibited the highest expression levels in the Basal and Normal subtypes. This observation suggests a compelling inference that CASC8 and LINC02188 may exert inhibitory effects on apoptosis by suppressing the expression of apoptosis-related proteins within the Basal and Normal subtypes.

**Comment 4:** **The patient data that has been used, does it also contain patients that have undergone therapy or have had a relapse? If yes, then how does this information fit with the prediction model?**

**Response**: Thank you for your suggestions. The data utilized in this investigation was sourced from the TCGA database. Notably, all patients underwent sampling without receiving any treatment at the time, and unfortunately, the database lacked information regarding patient treatment and recurrence. Subsequently, we conducted a comprehensive survival prognosis analysis using follow-up data, revealing that individuals with the Lum A subtype exhibited the most favorable five-year survival rate, standing at an impressive 89%, indicative of a more favorable prognosis. Currently, numerous documents have underscored the impact of drug treatment on the expression of lncRNA. For instance, in triple-negative breast cancer, Cisplatin has been reported to induce pyroptosis by upregulating lncRNA MEG3 (PMID: 34326697), while paclitaxel has been associated with the induction of drug resistance in breast cancer cells through the upregulation of lncRNA H19 (PMID: 33667788). Consequently, our future endeavors will involve the collection of additional clinical data to delve deeper into the intricate relationship between drug treatment and its potential influence on our model.

**Comment 5: Please include a table where all** **the changes associated that the authors have predicted with each of the 5 genes are listed. Please also include a comment section that contains authors' opinion on the prognosis and therapy based on their observations.**

**Response**: Thank you for your suggestions. We have incorporated a comprehensive table that succinctly summarizes all modifications pertaining to the five genes, accompanied by corresponding treatment recommendations. Here are the specific modifications:

**Table 2. Expression of key ncRNAs in breast cancer subtypes and relationships with RNA methylation, immunity, apoptosis and angiogenesis**

| ncRNAs | | *CASC8* | *GRIK1-AS1* | *LINC00511* | *ROCR* | *LINC02188* |
| --- | --- | --- | --- | --- | --- | --- |
| Subtype | Basal | 2.34 ± 1.4 | 0.15 ± 0.2  (lowest) | 1.72 ± 0.9 | 2.71 ± 1.6 | 3.73 ± 1.6  (highest) |
|  | Her2 | 0.39 ± 0.5 | 0.12 ± 0.1 | 0.68 ± 0.5  (highest) | 0.08 ± 0.1  (lowest) | 0.31 ± 0.6 |
|  | LumA | 0.37 ± 0.4 | 0.86 ± 0.9  (highest) | 0.36 ± 0.2  (lowest) | 0.51 ± 0.6 | 0.5 ± 0.6 |
|  | LumB | 0.27 ± 0.4 | 0.22 ± 0.3 | 0.36 ± 0.3  (highest) | 0.08 ± 0.1  (lowest) | 0.16 ± 0.5 |
|  | Normal | 0.95 ± 1.1 | 0.69 ± 0.8 | 0.66 ± 0.7 | 1.24 ± 1.6 | 1.4 ± 1.8  (highest) |
| RNA methylation | m1A | Positive correlation | Negative correlation | Positive correlation | Positive correlation | Positive correlation |
|  | m5C | Positive correlation | Negative correlation | Positive correlation | Positive correlation | Positive correlation |
|  | m6A | Positive correlation | Negative correlation | Positive correlation | Positive correlation | Positive correlation |
| Immune | checkpoint molecules | Positive correlation | Negative correlation | Positive correlation | Positive correlation | Positive correlation |
|  | Immune cells  (Suppress tumor) | Positive correlation | Negative correlation | Positive correlation | Positive correlation | Positive correlation |
|  | Immune cells  (Promote tumor) | Negative correlation | Positive correlation | Negative correlation | Negative correlation | Negative correlation |
| angiogenesis | | Positive correlation | - | Positive correlation | Positive correlation | Positive correlation |
| apoptosis | | Negative correlation | - | Negative correlation | Negative correlation | Negative correlation |

Upregulation of GRIK1-AS1 has been observed to decrease the level of RNA modification in both Basal and Her2 subtypes. Targeting the reduction of LINC00511 results in a decrease in the level of RNA modification in Her2 and LumB subtypes. Specifically targeting the reduction of ROCR has been found to reduce the level of RNA modification in the Basal subtype. Moreover, the targeted reduction of LINC02188 has been associated with an enhancement of angiogenesis in both Basal and Normal subtypes.

**Comment 6: Are there any plans to test these genes for validation in the laboratory?**

**Response**: Thank you for your suggestions. This study developed a predictive model for breast cancer subtypes utilizing a random forest algorithm and successfully identified five biomarkers associated with these subtypes. Simultaneously, a robust correlation was observed between key ncRNA expressions, the immune microenvironment, RNA methylation, and angiogenesis. Our findings offer detailed molecular insights into the predictive role of cuproptosis in determining breast cancer subtypes. Validating the significance of key ncRNAs in these subtypes holds promise for the enhanced application of cuproptosis-related ncRNAs in clinical settings. The database primarily comprises breast cancer patients of Caucasian and African-American descent from North America. Currently, we are in the process of collecting breast cancer subtype samples for validation, with a specific focus on the Chinese population. However, due to the time-consuming nature of sample collection and the intricacies involved in experimental procedures to verify the role of key genes, this article presents only the results of existing bioinformatics analyses. Here are the specific modifications:

- On Page 24, Line 528:

Furthermore, the verification of key genes' roles in breast cancer subtypes necessitates intricate experimental procedures due to the extended duration required for collecting samples of these subtypes. Consequently, this article solely encompasses the present bioinformatics analysis results. Our future endeavors involve substantiating the association between the cuproptosis-related prediction model and breast cancer subtypes through rigorous in vivo or in vitro experiments. Additionally, we aim to delve into the potential mechanisms underlying its impact on breast cancer, thereby enhancing the reliability of our research findings.

**Comment 7: What is the publicly available literature on cuproptosis and breast cancer?**

**Response**: Thank you for your suggestions. We employed PubMed as our primary resource to access a multitude of public documents pertaining to cuproptosis and its association with breast cancer. In our article, we meticulously referenced pertinent documents to substantiate our research findings and enhance the overall credibility of our work. Here are the specific modifications:

- On Page 5, Line 74:

Prior investigations have demonstrated that a constructive breast cancer prognosis prediction model can be developed by scrutinizing the involvement of cuproptosis-related genes in breast cancer prognosis. This entails a thorough examination of the correlation between cuproptosis-related genes and crucial factors such as tumor microenvironment and clinical characteristics (references: 36059516, 36640225). Furthermore, the predictive utility of cuproptosis-related genes extends to forecasting drug sensitivity in triple-negative breast cancer patients (references: 36341760, 37529698). Notably, cuproptosis has been identified as a contributor to c-Myc-mediated breast cancer stemness, as highlighted in a recent study (PMID: 37353799).

**Comment 8: The authors have looked at multiple aspects of cuprotosis related genes in tumor progression. While this is great, this also goes against them as they tried to look at too many things and not validated even a single phenomenon in the wet lab. Please discuss and comment.**

**Response**: Thank you for your suggestions. This study delved into the viability of targeting cuproptosis-related ncRNA as a therapeutic approach. The investigation revealed significant associations between RNA methylation, the tumor microenvironment, angiogenesis, and key genes. Moreover, it was observed that RNA methylation and the tumor microenvironment jointly influence angiogenesis. Consequently, the exploration of multiple factors aims to enhance our understanding of angiogenesis in distinct subtypes of breast cancer, laying a valuable foundation for diagnostic and personalized treatment research in breast cancer patients. Regrettably, the acquisition of breast cancer subtype samples is a time-consuming process, and validating the role of key genes in these subtypes necessitates intricate experimental procedures. Consequently, this article presents the current findings derived from bioinformatics analysis. Future plans include conducting in vivo or in vitro experiments to further validate the model's relevance to breast cancer subtypes and undertaking comprehensive investigations into its potential mechanisms within breast cancer. These efforts aim to enhance the overall reliability of the research results.

**Comment 9: Also, there is no effort in linking different parameters like tumor microenvironment, methylation, angiogenesis. Please include some sentences. Response**: Thank you for your suggestions. We have intricately connected the three key parameters tumor microenvironment, RNA methylation modification, and angiogenesis to engage in a comprehensive discussion. Here are the specific modifications:

- On Page 20, Line 423:

It is well-established that the development of new blood vessels plays a pivotal role in the progression of solid tumors, serving as a crucial precursor for tumor invasion and metastasis (PMID: 19818284). Recent investigations have revealed that IGF2BP2 has the capability to infiltrate endothelial cells via exosomes, thereby fostering angiogenesis and metastasis in LUAD (PMID: 37353784). METTL14, through the modulation of TGFβ via the RhoA and PI3K-AKT pathways, contributes to tumor angiogenesis and progression (PMID: 30306128). Additionally, YTHDF3 has been identified as a promoter of cancer cell-astrocyte interaction, thereby facilitating angiogenesis. Notably, current research underscores the therapeutic potential of targeting METTL3 to combat angiogenesis in bladder cancer (PMID: 33618740).

Furthermore, tumor neovascularization is implicated in the secretion of angiogenic factors that suppress the activity of cytotoxic T cells, culminating in tumor immunosuppression (30742782). Concurrently, tumor cells or macrophages within the tumor microenvironment release substantial quantities of pro-angiogenic factors such as VEGF, bFGF, and PDGF. These factors activate pro-angiogenic signaling pathways, fostering the growth, invasion, and metastasis of tumor blood vessels (PMID: 32993787). Collectively, these studies underscore the role of RNA methylation and the tumor microenvironment in promoting tumor angiogenesis, presenting them as potential targets for cancer treatment.

As indicated in Table 2, this study identified high expression of LINC02188 in the Basal subtype and elevated expression of GRIK1-AS1 in LumA. Notably, five cuproptosis-related ncRNAs demonstrated a significant positive correlation with RNA methylation, angiogenesis factors, and immune cells. This correlation suggests that in the Basal subtype, LINC02188 may enhance the release of tumor angiogenesis factors (TNFRSF21, PRG2) by augmenting RNA methylation, consequently promoting angiogenesis. Similarly, in the LumA subtype, GRIK1-AS1 might mitigate cancer malignancy by inhibiting T cell and B cell activity, thereby reducing the extent of RNA methylation modification.

**Comment 10: The analysis is subtype specific. What about the changes associated with stage, therapy etc. Please include this information.**

**Response**: Thank you for your suggestions. Breast cancer exhibits diverse TMN grades and clinical stages, and our study sought to investigate the expression of key genes across these variations. Utilizing box plots, we aimed to delineate disparities in gene expression among different T grades and clinical stages of breast cancer. Surprisingly, our findings revealed no statistically significant differences in the expression of these key genes across distinct stages and T grades (P > 0.05). Consequently, we postulate that the identified key genes may not be correlated with the clinical stage and T grade of breast cancer.

Moreover, the majority of patients, approximately 2/3, presented with breast cancer TMN grades of M0 and N0. This skewed data distribution posed challenges, impeding the precise determination of key gene expression in various M and N stages. Consequently, our investigation could not ascertain the relationship between cuproptosis and M or N grading. Furthermore, none of the patients underwent any form of treatment, preventing us from exploring potential associations between cuproptosis and therapeutic interventions.

Despite these limitations, our commitment to advancing research remains unwavering. Future endeavors will involve the ongoing collection of additional clinical samples, allowing us to delve deeper into treatment-related changes and further unravel the intricacies of key gene expression in breast cancer.

**Comment 11: Please include a graphical abstract summarizing the findings and the take home message with potential use in disease prognosis.**

**Response**: Thank you for your suggestions. Here are the specific modifications:

**Fig. 1. The flow diagram of data collection and analysis in the present study**

Finally, we appreciate for Editors/Reviewers’ warm work earnestly, and hope that the correction will meet with approval. Once again, thank you very much for your comments and suggestions.

Best wishes,

Yours sincerely,

Shiwei Duan, Ph. D, Professor

School of Medicine,

Hangzhou City University,

Zhejiang, China

Email: duansw@hzcu.edu.cn
